# Exploring the Spatial Distribution of Rheumatic Diseases and Its Correlation With Temperature and Humidity Among Middle-Aged and Elderly Adults in China

**DOI:** 10.3389/ijph.2022.1604782

**Published:** 2022-07-21

**Authors:** Yaofeng Han, Qilin Sheng, Ya Fang

**Affiliations:** State Key Laboratory of Molecular Vaccinology and Molecular Diagnostics, School of Public Health, Xiamen University, Xiamen, China

**Keywords:** temperature, spatial regression model, relative humidity, middle-aged and elderly adults, rheumatic diseases

## Abstract

**Objectives:** This study aimed to analyze the prevalence of rheumatic diseases and its correlation with temperature and humidity among middle-aged and elderly adults in China from a spatial perspective.

**Methods:** Data on rheumatic diseases among middle-aged and elderly adults were sourced from the 2018 China Health and Retirement Longitudinal Study (CHARLS). Moran’s *I* was applied to explore the spatial autocorrelation of rheumatic diseases. Spatial lag model (SLM) was established to probe the correlation between rheumatic diseases and temperature and humidity.

**Results:** The age-standardized prevalence of rheumatic diseases was 33.2% for middle-aged and elderly adults in China, varying from 12.0% to 51.4% depending on regions. The Global Moran’s *I* was 0.506 (*p* = 0.001). Average temperature had negative correlation while average relative humidity had positive correlation with age-standardized prevalence of rheumatic diseases in the SLM.

**Conclusion:** The age-standardized prevalence of rheumatic diseases of middle-aged and elderly adults showed spatial autocorrelation in China. We recommend taking measures to prevent rheumatic diseases for the middle-aged and elderly adults, especially for those living in cold and humid regions.

## Introduction

Rheumatic diseases are autoimmune and inflammatory diseases that include a wide range of painful bone and joint diseases, surrounding soft tissue diseases, and systemic medical conditions, such as osteoarthritis (OA) and rheumatoid arthritis (RA) [1]. Rheumatic diseases are characterized by unclear pathogenesis, insidious and slow onset, and longer course of disease [2].

According to previous studies, rheumatic diseases have a high prevalence and disability rate, which brings a heavy disease burden to individuals, families, and society [3, 4]. Globally, the age-standardized prevalence of RA is 246.6 per 100,000 persons, accounting for 3.4 million disability adjusted life years (DALYs) [5]. There were about 303.1 million OA cases, with an age-standardized prevalence of 3,754.2 per 100,000 persons. Nearly 9.6 million years lived with disability (YLDs) were due to OA within the globe [6]. A survey in China conducted by Professional Committee of Rheumatic Diseases, Chinese Association of Integrative Medicine showed that the prevalence of rheumatic diseases in the whole population was 17.4% [7]. Furthermore, rheumatic diseases also have a high disability rate due to the lack of relevant health knowledge of rheumatic diseases [8]. The 2-year disability rate for RA patients without treatment was 50%, while the 3-year disability rate was as high as 70% [9]. The physically disabled population caused by OA in China was about 4.43 million, accounting for 18.38% of the total physically disabled population according to the results of the second Chinese national survey on disability [10]. The burden of RA patients expressed in cost of illness was USD $3,826 per capita per year in China—an amount that is comparable with the costs reported in Japan [11, 12]. Therefore, rheumatic diseases are regarded as an important public health issue in China.

In previous studies, gender and education attainment played an important role in contributing the cause of rheumatic diseases [13, 14]. Women and people with lower education attainment were at high risk of developing rheumatic diseases. Marital status, reflecting the influence of social support, was also associated with rheumatic diseases [15]. Several studies had evaluated the association between rheumatic diseases, temperature, and humidity; however, the findings vary. Temperature had an established negative correlation with rheumatic diseases while relative humidity had a positive correlation with rheumatic diseases [16, 17]. However, one study provided evidence against an association between rheumatic diseases and average temperature and average relative humidity [18].

The prevalence of rheumatic diseases are significantly regionally heterogeneous [19, 20]. The Global Moran’s *I* for RA in Alberta, Canada was 0.15 (*p* < 0.05); compared with the metro areas (RR: 10.69), a higher rate was observed in rural areas (RR: 14.46) [21]. Geographical analysis of country clustering showed significant variation across France, with lower prevalence in the northwest and higher prevalence in the southeast area (*p* = 0.012) [22]. These studies suggested that conducting research on rheumatic diseases from a spatial perspective is warranted. However, classical statistical methods cannot analyze the spatial characteristics of diseases due to the dependence of samples. Therefore, spatial regression models were proposed and developed rapidly. Spatial regression model, taking full account of spatial autocorrelation and spatial heterogeneity, had become an important method to study the spatial characteristics and influencing factors of diseases [23, 24].

This study intended to explore the prevalence of rheumatic diseases among middle-aged and elderly adults in China and to examine its correlation with temperature and humidity from a spatial perspective. We hope to provide scientific reference for comprehensive prevention and control of rheumatic diseases.

## Methods

### Study Area

The People’s Republic of China is located in the east of Asia between 73°33′E and 135°05′E longitude, and 3°51′N and 53°33′N. The total area is 9.6 million km^2^ with a population of over 1.4 billion. The climate of China is complex, diverse, and differs geographically—varying between tropical, subtropical, warm temperate, middle temperate, cold temperate, and Qinghai-Tibet plateau climate.

### Data Source

The data on rheumatic diseases sourced from the 2018 China Health and Retirement Longitudinal Study (CHARLS), which covered 28 provinces (cities and autonomous regions) and 23,000 participants, were widely used and recognized in academia. According to the age classification standards issued by the World Health Organization (WHO), the middle-aged and elderly adults in this study were people aged 45 and older. The proportion of illiterate population was used to represent education attainment. Illiterate refers to no formal education. The proportion of living with a partner was used to represent the marital status. The above data were all from CHARLS.

Average temperature and average relative humidity data were obtained from China Health Statistical Yearbook.

### Statistical Analysis

#### Descriptive Analysis

The missing values in rheumatic disease data were supplemented by recalling the follow-up information of research subjects in the CHARLS 2013 and 2015 data. Using the 2010 Chinese population census data for age standardization, we calculated the age-standardized prevalence and drew a spatial distribution map to describe the prevalence and regional differences of rheumatic diseases.

#### Spatial Autocorrelation Analysis

In order to explore the information about spatial autocorrelation, we calculated the Moran’s *I* for rheumatic diseases, average temperature, and average relative humidity. Local indicators of spatial association (LISA) cluster map were used to explore the spatial cluster types and specific cluster location of diseases in the study area.

#### Spatial Regression Analysis

Firstly, the ordinary least squares (OLS) model was used to explore the correlation between average temperature, average relative humidity, and the age-standardized prevalence of rheumatic diseases. The residuals of the OLS model were tested by Moran’s *I* to determine whether spatial regression models should be performed and the Lagrange Multiplier test was used to select an optimal model from the spatial lag model (SLM) and spatial error model (SEM). If the Moran’s *I* of residuals of the OLS model shows spatial autocorrelation, the spatial regression models were used. Then, if the Lagrange Multiplier-error is statistically significant, the SEM would be selected; and if the Lagrange Multiplier-lag is statistically significant, the SLM would be selected.

Secondly, the spatial regression model (SLM or SEM) was applied to fit disease data, and the results were compared with that of the OLS model. The level of statistical significance was defined as *p* ≤ 0.05. The OLS model takes the following form:

Y=α+βX+ε (1)

The general form of spatial lag model (SLM):

$$\text{Y}=\text{ρ}W_{1}Y+\text{βX}+\text{ε,}\;\;\;\;\;\;\text{ε}∼\text{N}\left(0,\sigma^{2}I_{n}\right)$$ (2)

The general form of spatial error model (SEM):

$$\text{Y}=\text{βX}+\text{ε,}\;\;\;\;\text{ε}=\text{λ}W_{2}\varepsilon +\mu ,\;\;\;\;\mu ∼N\left(0,\sigma^{2}I_{n}\right)$$ (3)

where Y is the age-standardized prevalence of rheumatic diseases; *α* is an intercept; *β* is the regression parameters; X is the independent variables, including average temperature, average relative humidity, the proportion of men, illiteracy rate and the proportion of living with partner; *ε* is the random error term; *W*_1_ and *W*_2_ are the spatial weights; *W*_1_ *Y* is the spatial lag term; *ρ* is the spatial autoregressive parameter of *W*_1_ *Y*; *W*_2_ *ε* is the spatial error term; *λ* is the autoregressive coefficient of *W*_2_ *ε*; and errors in *μ* are individually independent and obeying normal distribution.

### Analysis Software

IBM SPSS 25.0 was used for descriptive analysis of the age-standardized prevalence of rheumatic diseases. ArcGIS 10.6.1 was used for spatial autocorrelation analysis and drawing the spatial distribution map. GeoDa 1.14.0 was used for spatial regression analysis.

## Results

### Descriptive Analysis

In 2018, the age-standardized prevalence of rheumatic diseases among middle-aged and elderly adults in China was 33.2%, and varied with provincial-level regions from 12.0% to 51.4% (Table 1).

**TABLE 1:** Age-standardized prevalence of rheumatic diseases among middle-aged and elderly adults in China (China, 2018).

Province	Age-standardized prevalence (%)		
	Men	Women	Total
Shanghai	6.5	14.0	12.0
Beijing	13.7	23.2	17.7
Shandong	17.9	25.1	21.7
Zhejiang	19.5	25.4	22.6
Liaoning	21.3	25.7	23.6
Jiangxi	23.5	37.2	23.6
Shanxi	20.4	27.1	23.7
Tianjin	15.4	33.8	24.4
Henan	24.2	26.0	25.2
Guangdong	21.2	32.2	26.6
Jiangsu	25.9	31.2	28.6
Hebei	25.5	33.7	29.7
Fujian	25.1	32.6	29.7
Jilin	26.7	34.5	30.9
Anhui	25.4	36.7	31.4
Gansu	36.7	41.9	32.1
Shan’xi	27.5	35.3	32.1
Guangxi	25.7	37.8	32.2
Mongolia	28.6	41.6	34.7
Heilongjiang	29.5	44.0	36.5
Guizhou	38.7	36.8	37.2
Xinjiang	32.4	40.4	38.6
Hunan	40.3	48.4	44.3
Qinghai	28.8	49.5	44.6
Hubei	39.9	48.1	44.9
Yunnan	40.5	55.0	48.0
Chongqing	38.6	57.5	50.9
Sichuan	43.5	58.4	51.4
Total	28.3	37.7	33.2

**FIGURE 1:**
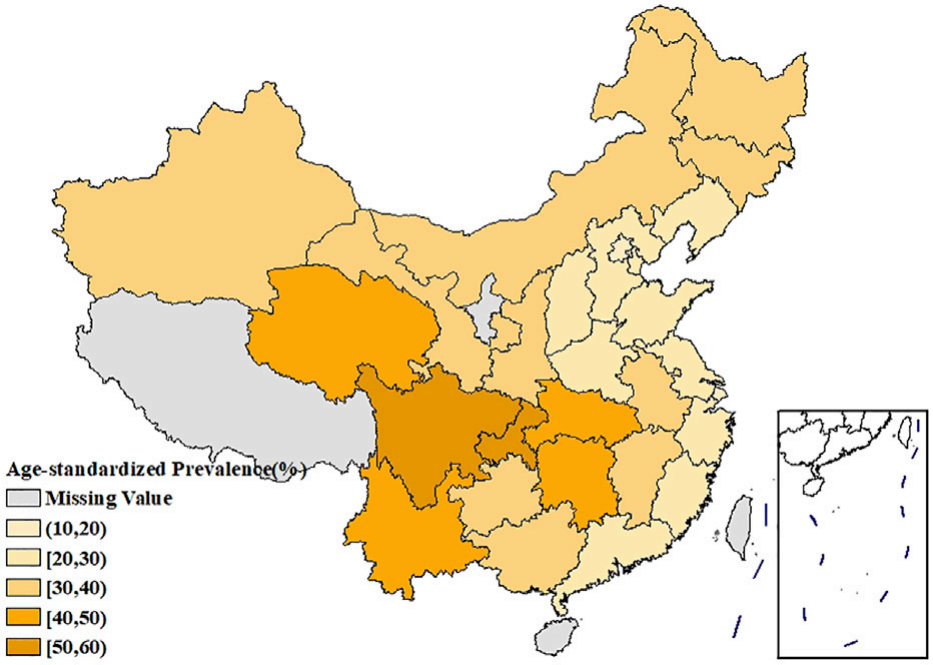
Spatial distribution of the age-standardized prevalence of rheumatic diseases in China (China, 2018).

**FIGURE 2:**
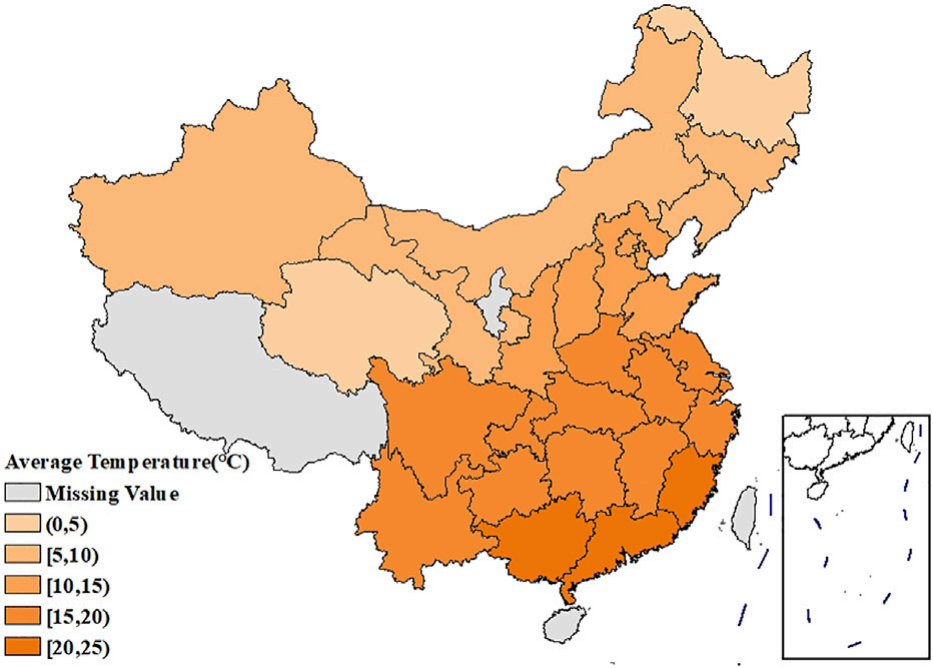
Spatial distribution of the average temperature in China (China, 2018).

**FIGURE 3:**
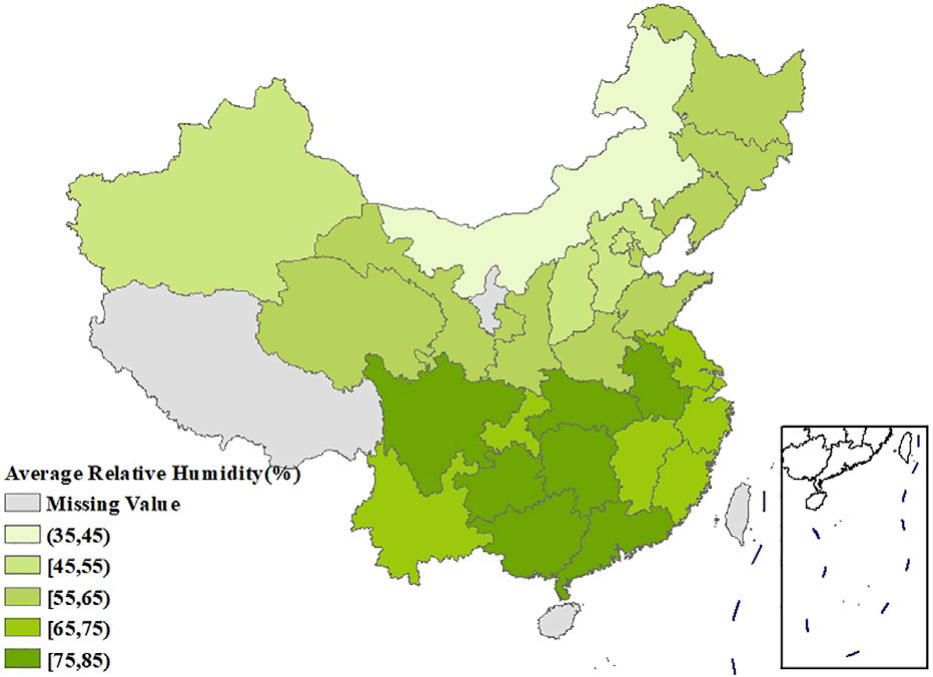
Spatial distribution of the average relative humidity in China (China, 2018).

Figure 1 showed the spatial distribution of the age-standardized prevalence of rheumatic diseases. It could be seen that the southwest China had the highest prevalence, with the prevalence in the surrounding regions gradually decreasing. Supplementary Figures S1, S2 showed that the age-standardized prevalence of rheumatic diseases in women had a similar spatial distribution pattern as in men, and the prevalence in each region was higher than that in men. The spatial distribution of average temperature and average relative humidity in China showed a trend of increasing gradually from north to south, as shown in Figures 2, 3.

### Spatial Autocorrelation Analysis

**FIGURE 4:**
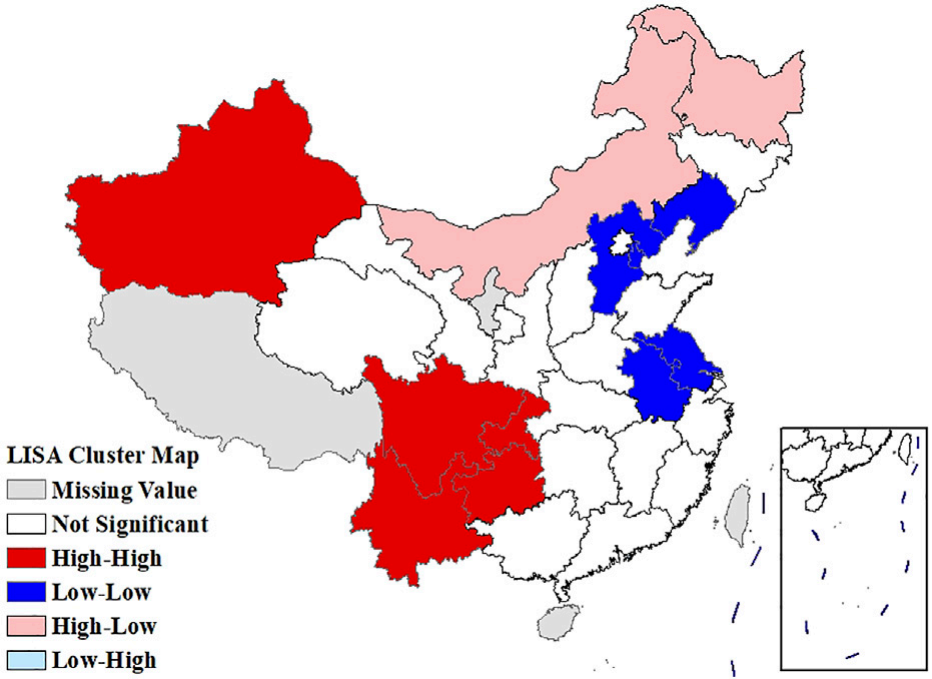
Local Moran’s *I* analysis (LISA) cluster map of the age-standardized prevalence of rheumatic diseases (China, 2018).

The global Moran’s *I* for the age-standardized prevalence of rheumatic diseases, average temperature, and average relative humidity were 0.506 (*p* = 0.001), 0.763 (*p* = 0.001), and 0.705 (*p* = 0.001), respectively, indicating the existence of spatial autocorrelation at the provincial scale. The LISA cluster map showed significant core clustering of high-high, low-low, and high-low of the age-standardized prevalence of rheumatic diseases (Figure 4).

### Regression Analysis

Statistical results of the OLS model (Table 2) demonstrate that the average temperature (*p* = 0.041) had a significantly negative correlation with the age-standardized prevalence of rheumatic diseases. However, the global Moran’s *I* for the residual of OLS model was 0.195, indicating the existence of spatial autocorrelation and a necessity for spatial regression analysis. We then applied SLM instead of SEM according to the results of Lagrange Multiplier test (*P*_Lagrange Multiplier-error_ = 0.149, *P*_Lagrange Multiplier-lag_ = 0.002).

**TABLE 2:** Results of the OLS model and SLM (China, 2018).

Variables	OLS		SLM	
	Coefficient	*p-*value	Coefficient	*p-*value
*ρ*	—	—	0.72	0.001
Intercept	91.0	0.169	96.7	0.016
AT	−1.0	0.041	−0.7	0.013
ARH	0.4	0.072	0.3	0.046
IR	0.3	0.060	0.2	0.015
PM	−1.7	0.190	−2.1	0.012
PLP	0.5	0.327	0.1	0.884
*R* ^2^	0.421	—	0.710	—
LLR	−96.126	—	−88.805	—
AIC	204.253	—	191.611	—
Moran’s *I* ^a^	0.195	0.008	−0.092	0.372

a The residual of model.

OLS, ordinary least squares model; SLM, spatial lag model, the dependent variable for all was age-standardized prevalence of rheumatic diseases. AT, average temperature; ARH, average relative humidity; IR, illiteracy rate; PM, proportion of men; PLP, proportion of living with partner; *ρ*, spatial autoregressive parameter; LLR, log-likelihood ratio; AIC, Akaike information criterion.

The average relative humidity (*p* = 0.046) and illiteracy rate (*p* = 0.015) had a significantly positive correlation with the age-standardized prevalence of rheumatic diseases, while the average temperature (*p* = 0.013) and the proportion of men (*p* = 0.012) had a significantly negative correlation according to the results of SLM. The global Moran’s *I* for the residual of SLM was −0.092, indicating that there was no spatial autocorrelation (Table 2).

The SLM showed greater *R* ^2^, log-likelihood and a smaller AIC value compared to the OLS model, which indicated that SLM was better than OLS as a model for rheumatic diseases (Table 2).

## Discussion

This study explored the prevalence of rheumatic diseases among middle-aged and elderly adults in China and its correlation with temperature and humidity using a spatial regression model based on follow-up data from 28 provinces (cities and autonomous regions) from CHARLS 2018. We found that the age-standardized prevalence of rheumatic diseases is spatially autocorrelated and heterogenous. Gender, education attainment, marital status, and humidity were not correlated with age-standardized prevalence of rheumatic diseases in the results of the OLS, which were inconsistent with other studies [13–15, 17]. The global Moran’s *I* for the residual of OLS was 0.195 (*p* = 0.008). It indicated that the residual was not independent and does not meet the analysis conditions of OLS [24]. The SLM revealed that age-standardized prevalence of rheumatic diseases was positively correlated with average relative humidity and illiteracy rate while negatively correlated with average temperature and the proportion of men; this observation was consistent with other studies [13, 14, 16, 17]. The global Moran’s *I* for the residual of SLM was −0.092 (*p* = 0.372). It demonstrated that SLM could analyze the spatial autocorrelation of rheumatic diseases by constructing the spatial lag term of the dependent variable [24].

The age-standardized prevalence in Sichuan and Chongqing was the highest at above 50%, while the age-standardized prevalence in Beijing and Shanghai was the lowest at less than 20%. In terms of spatial distribution, with Sichuan and Chongqing as the center, the prevalence in the surrounding regions gradually decreased. LISA cluster map showed that high-high clustering model of rheumatic disease was distributed in Xinjiang, Sichuan, Chongqing, Yunnan, and Guizhou. The prevalence of rheumatic diseases was high in these regions and their adjacent regions. The high-low clustering model of rheumatic disease was distributed in Inner Mongolia and Heilongjiang. The prevalence of rheumatic diseases in these regions was high but low in the adjacent regions. The low-low clustering model of rheumatic disease was distributed in Liaoning, Beijing, Jiangsu, Anhui, and Hebei. The prevalence of rheumatic diseases was low in these regions and their adjacent regions. A similar distribution pattern of rheumatic diseases was revealed in several studies. This may be related to the extensive area with very complex climate and environment in China [4, 25]. Our research found that the age-standardized prevalence of rheumatic diseases was negatively correlated with average temperature, which was consistent with the results reported by Zeng et al. [26]. We also found direct positive correlation between average relative humidity and rheumatic disease. High humidity can aggravate rheumatic disease by increasing joint stiffness [27]. Emerging evidence suggests that microorganisms are involved in the pathogenesis of rheumatic diseases [28]. Pathogenic microorganisms will grow and reproduce quickly in a highly humid environment, so this may also aggravate rheumatic disease. The spatial distribution of average temperature and average relative humidity in China showed a gradually increasing trend from north to south. The prevalence was higher in tropical and subtropical regions such as Sichuan and Chongqing, which are located in mountainous areas with difficult access. The higher prevalence could be due to the higher relative humidity in the air, lower level of economic development, and more limited health resources. Although the middle temperate and cold temperate zones such as Inner Mongolia and Northeast China had the lowest average temperature in the whole of China, the age-standardized prevalence was not high. This may be because these regions have the appropriate indoor temperature benefiting from proper heating facilities and low air humidity. The eastern coastal areas of China have lower prevalence because of the relatively developed economic conditions and abundant medical and health resources, which offset the partial influence of relative humidity. China has a complex geographical area and diverse populations. Genes, living habits, and folk culture may lead to different populations being more susceptible to temperature and humidity, and thus more susceptible to rheumatic diseases. This may also be the reason for the large differences in the spatial distribution of rheumatic diseases. The prevalence of rheumatic diseases was at a high level and there were large regional differences in China. Therefore, we should invest more healthcare resources and allocate reasonably, focusing on high-prevalence areas such as Sichuan, Chongqing, and Yunnan. We should also be using spatial thinking and combining the spatial distribution characteristics of rheumatic diseases to formulate region-specific measures to reduce the prevalence and disease burden of rheumatic diseases.

Additionally, the illiteracy rate (*p* = 0.015) had positive correlation with the age-standardized prevalence of rheumatic diseases. Most of the population with lower education attainment lack sufficient awareness of rheumatic diseases and do not pay enough attention to their own health conditions, which may promote the occurrence of rheumatic diseases [14, 29]. Meanwhile, our study had pointed out a relationship between education attainment and socioeconomic status. People with lower education attainment tend to have lower socioeconomic status and less access to healthcare services and medications; thus this fact may affect disease progression [30, 31]. The proportion of men (*p* = 0.012) had negative correlation with the age-standardized prevalence of rheumatic diseases. Estrogens can stimulate humoral immune responses while androgens can exert suppressive effects on both humoral and cellular immune responses, making women more susceptible to rheumatic diseases. The differences in physiological structure and lifestyle habits may also lead to higher risk for women, a fact that was consistent with the conclusions drawn by Falsarella et al. [32]. At present, women are still in a relatively disadvantaged position and at higher risk of developing rheumatic diseases. We recommend paying more attention to women’s health to promote the process of medical fairness. Improve the health awareness of population and the understanding of rheumatic diseases by combining new media to formulate propaganda methods adapted to different people.

Relying on big data and artificial intelligence technology, we can acquire large-scale and complex disease data. Research proves that these disease data are spatially autocorrelated, rather than simply assuming they are independent of each other. When we take into account the spatial autocorrelation, apparently, some traditional statistical analysis methods are no longer applicable. In this paper, we used SLM and OLS models to research the prevalence of rheumatic diseases, respectively. SLM with the larger *R* ^2^ (0.710 vs. 0.421) and smaller AIC (191.611 vs. 204.253) performed better than OLS model. In addition, these factors, considered to be related to rheumatic diseases in previous research, were still discovered in SLM but not statistically significant in OLS. The coefficient *ρ* of the spatial lag term in SLM was 0.72 (*p* = 0.001), indicating that changes in these factors will not only influence the prevalence of rheumatic diseases in this region (the direct effect), but also influence the prevalence of adjacent regions (the indirect effect). Therefore, we have reason to believe that spatial analysis methods like SLM can provide a new perspective for the current research on disease distribution and influencing factors, from which more disease information can be discovered.

There are limitations that ought to be acknowledged in this study. Firstly, the data that we obtained from CHARLS 2018 were from self-reported cases without further verifying the types of rheumatic diseases, this may have biased the prevalence preventing the analysis of specific types of rheumatic diseases. Secondly, the data of annual temperature and average relative humidity did not fully reflect the real regional differences in environmental factors. Thirdly, our study was a cross-sectional study that explored the group correlations, ignoring the effects of time factors and individual susceptibility to rheumatic diseases. Fourthly, we were unable to analyze the relationship between rheumatic diseases and other factors such as BMI, occupation, and others because of the data availability. Lastly, SLM assumed that the effect of independent variables on rheumatic diseases was consistent for all regions, which ignored the spatial non-stationarity. Thus, in further research, a local spatial regression model, such as geographically weighted regression model (GWR), could be used to explore the local effects of influencing factors.

This study provided comprehensive evidence that the age-standardized prevalence of rheumatic diseases had spatial autocorrelation. The exploration of spatial regression predictors identified that the age-standardized prevalence of rheumatic diseases was positively correlated with the average relative humidity, while negatively correlated with the average temperature. These findings can provide a reference to facilitate better understanding of prevalence and to make more effective strategies to control rheumatic diseases. Policies such as strengthening publicity and education on rheumatic diseases and maintaining the air humidity and temperature at an appropriate level in living and working environments should be implemented to reduce the burden on bones, joints, and muscles. We should pay more attention to, and increase the allocation of health resources for, regions with a high prevalence of middle-aged and elderly adults with a high risk of rheumatic diseases.

## Supplementary Material

Supplementary Figure S1 Spatial distribution of the age-standardized prevalence of rheumatic diseases in men (China, 2018).

Supplementary Figure S2 Spatial distribution of the age-standardized prevalence of rheumatic diseases in women (China, 2018).
