# Supplementary figures and images for: Exploring the Spatial Distribution of Rheumatic Diseases and Its Correlation With Temperature and Humidity Among Middle-Aged and Elderly Adults in China
Source: Int J Public Health. 2022 Jul 21;67:1604782. doi: 10.3389/ijph.2022.1604782 (PMC9351402; doi:10.3389/ijph.2022.1604782)

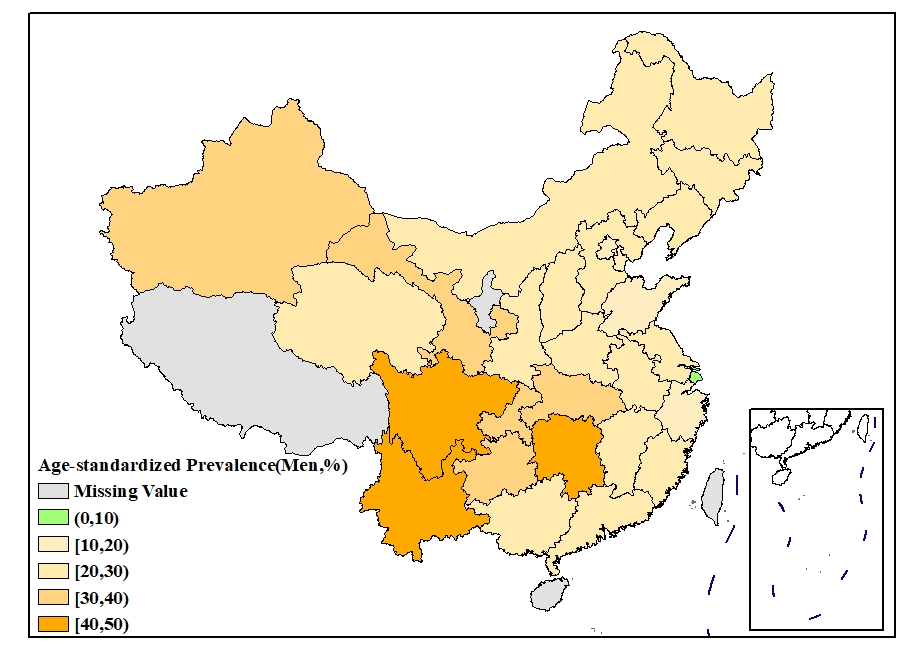

Supplement: Supplementary file 1 [file Image1.jpeg]

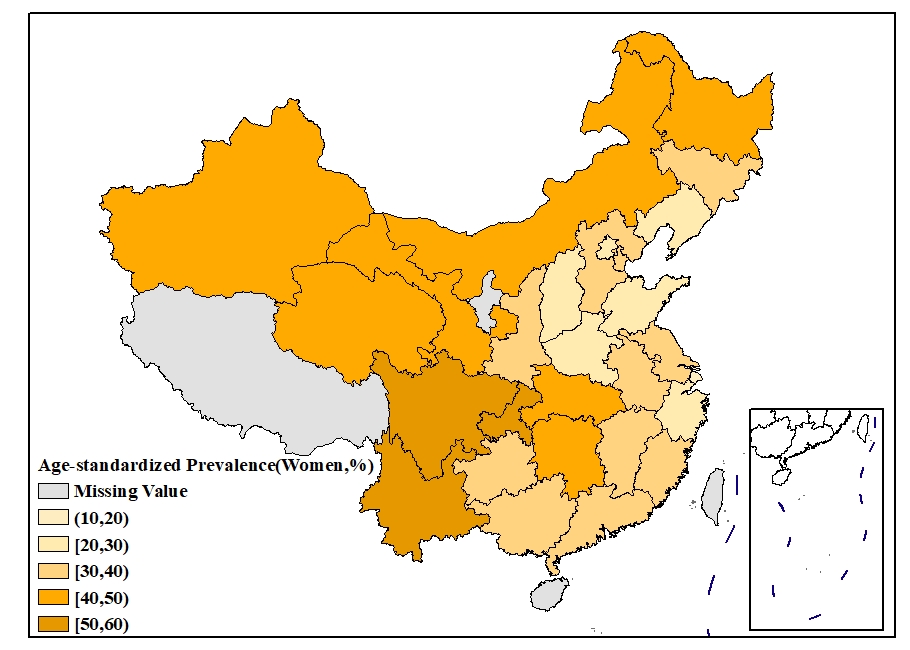

Supplement: Supplementary file 2 [file Image2.jpeg]
